# Photobiomodulation effects on synovial morphology, iNOS gene, and protein expression in a model of acute inflammation

**DOI:** 10.1590/acb392024

**Published:** 2024-03-15

**Authors:** Fernando Russo Costa do Bomfim, Bruna Silva Gomes, Sabrina Zanchetta Lanza, Marcelo Augusto Marretto Esquisatto, Gaspar de Jesus Lopes-Filho

**Affiliations:** 1 Universidade Federal de São Paulo – Escola Paulista de Medicina – Postgraduate Program in Interdisciplinary Surgical Science – São Paulo (SP), Brazil.; 2 Centro Universitário da Fundação Hermínio Ometto – Araras (SP), Brazil.

**Keywords:** Low-Level Light Therapy, Inflammation, Nitric Oxide Synthase Type II

## Abstract

**Purpose:**

To evaluate morphological aspects and inducible nitric oxide synthase (iNOS) gene and protein expression in a model of acute inflammation.

**Methods:**

Thirty-six female Wistar rats were assigned into three groups: control (saline, n = 12), sham (arthritis, n = 12), and PBM (arthritis and photobiomodulation, n = 12). Arthritis induction was performed with 200 μg of intra-articular Zymosan in sham and PBM animals. PBM was performed 24 h after induction with a laser device (λ = 808 nm, 25 mW of nominal power, fluence of 20 J/cm^2^, beam area of 0.02 mm^2^, time of 33 s, total energy of 0.825 J) with punctual and single dose application. Morphological analysis of joint structure (HE) and immunohistochemistry (anti-iNOS antibody) were performed on knee samples, and synovial tissue was submitted to RNA extraction, cDNA synthesis and gene expression analysis by quantitative polymerase chain reaction. Statistical analyses were performed with p < 0.05.

**Results:**

It was observed an increase in the thickness of the synovial lining epithelium and inflammatory infiltrate in sham compared to PBM. Gene expression analysis showed higher iNOS expression in PBM, and iNOS protein expression decreased in PBM compared to sham.

**Conclusions:**

Photobiomodulation decreased inflammation in PBM animals, upregulated iNOS gene expression, however down egulated protein expression compared to sham.

## Introduction

Inducible nitric oxide synthase (iNOS) is an enzyme that plays a fundamental role in the biological system, involved in the modulation of cellular and physiological processes in response to pathological and inflammatory stimuli. iNOS belongs to the nitric oxide synthase family, a group of enzymes responsible for nitric oxide synthesis^1^^,^ 2.

In terms of its actions in the biological system, iNOS differs from other enzymes in its family in that, unlike the other constitutive nitric oxide synthase isoforms, it is produced under conditions of stress, inflammation, and tissue damage. Activation of iNOS occurs in response to a variety of stimuli, including pro-inflammatory cytokines, bacterial lipopolysaccharides, and inflammatory chemicals. Its induction results in significantly increased production of nitric oxide compared to constitutive isoforms. Nitric oxide produced by iNOS plays a critical role in the regulation of the immune system, control of blood flow, neurotransmission and cellular homeostasis^3^^,^ 4.

A precise balance between iNOS activity and nitric oxide production is extremely important for tissues, as high levels of iNOS and nitric oxide have been associated with pathological conditions such as cardiovascular disease, chronic inflammation, and cell damage. Therefore, understanding the mechanisms underlying the regulation of iNOS and the effects resulting from its excessive activation is fundamental for understanding the pathophysiology of various diseases and for exploring targeted therapeutic strategies^5^^,^ 6.

iNOS plays an important role in arthritis and inflammatory joint diseases. In diseases such as rheumatoid arthritis and osteoarthritis, iNOS activation and subsequent nitric oxide production are closely linked to the inflammatory process and disease progression, because iNOS production occurs in synovial cells^7^.

In synovial cells, activation of iNOS triggers severe morphologic responses, as it is associated with an increase in nitric oxide production, which contributes to an increase in synovial vascularization and favors a marked cellular infiltration, intensifying the local inflammatory response. The action of iNOS on synoviocytes can also lead to a significant increase in edema, compromising the structure and function of the synovial membrane, which shows intense leukocyte infiltration, synovial thickening, synovial fibrosis and contributes to joint degradation^7^.

The progression of arthritis involves joint damage associated with the release of iNOS, which causes damage to joint and periarticular tissues, resulting in cartilage degradation and activation of inflammatory processes in a cycle of sustained tissue damage leading to permanent joint complications^6^^,^ 8.

Given the link between iNOS, nitric oxide and inflammation in joint disease, the regulation of iNOS has been the subject of therapeutic research using selective iNOS inhibitors and inflammation modulators with the goal of slowing the progression of arthritis^8^.

Among possible anti-inflammatory methods, photobiomodulation (PBM) with low-level laser is a non-invasive therapy that uses light with anti-inflammatory and analgesic properties to improve the course of acute and chronic inflammatory diseases such as arthritis^9^.

PBM with low-level laser therapy (LLLT) has been shown to increase tissue metabolism, reduce pain and inflammation, and accelerate the regeneration process. PBM has properties to increase cell viability, modify gene and protein regulation, and consequently modulate several biological processes including the reduction of synovial damage and joint degeneration^10^^,^ 11.

The aim of this study was to evaluate the effects of low-level laser PBM on synovial morphology and modulation of iNOS gene and protein expression in a model of acute joint inflammation.

## Methods

### Animals, experimental groups, and arthritis induction

This study was performed by Ethical Principles of the Brazilian College of Animal Experimentation and approved by Comissão de Ética em Uso Animal of the Centro Universitário da Fundação Hermínio Ometto, protocol no. 077/2017.

Thirty-six female Wistar rats were under light/dark cycle of 12 h and received water and food *ad libitum*. The animals were assigned into three groups:

- Control (saline intra-articular injection, n = 12);
- Sham (arthritis induction, n = 12);
- PBM (arthritis induction and PBM, n = 12).

Arthritis induction was performed with 200 μg of Zymosan intra-articular after an anesthetic plane (Ketamine [0.3 mg/kg]-Xylazine [0.1 mg/kg]) in sham and PBM groups, while the control one received saline solution as describe before by our group^12^.

### Photobiomodulation with low-level laser

Twenty-four h after the induction, group PBM received low-level laser application by a Galium Arsenide device (AsGa, DMC group, São Carlos, SP, Brazil) with λ = 808 nm, 25 mW of nominal power, fluence of 20 J/cm^2^, beam area of 0.02 mm^2^, time of 33 s, total energy of 0.825 J, with punctual application by the single-dose method. Control and sham were submitted to the same protocol with the device turned off^12^.

### Euthanasia and sample

Samples were collected from all groups seven days after induction. Animals were euthanized by deep anesthesia, and blood was collected by cardiac puncture. Synovial regions were collected after exposure of the joint capsule (n = 6 per group) and stored in RNA later solution to preserve RNA molecules for later extraction. Joint samples (n = 6 per group) were collected in buffered formalin and subjected to standard histologic processing (decalcification, dehydration, embedding, and sectioning) for subsequent morphologic and immunohistochemical analysis.

### Morphological analyses

For morphological analyses, qualitative data, knee samples were stained with hematoxylin and eosin, and it was performed evaluation of inflammatory areas in sinovia (μm^2^). Histological images were obtained from three digital images from each animal (n = 18 images/animal) documented from the middle region of the knee joint in each experimental group. The photodocumentation was performed using a Leica DFC300 FX microscope, and the images were analyzed using the Image J program (NIH/USA, free program).

### Gene expression and immunohistochemistry

RNA was extracted from all knee samples with a commercial kit AxyPrep Multisource Total RNA (Axygen, Tewksbury, MA, United States of America) according to manufacturer instructions. cDNA was synthesized from 20 ng of total RNA with Superscript III (2,000 U/μL, Invitrogen Technologies). Gene expression was performed by real time polymerase chain reaction technique after reverse transcription (RT-qPCR) for β-actin (Forward: 5’-TCCTAAGCCAGTGCCAGAAG-3’ and Reverse: 5’-TCATTCGTCTGTTTCCCATTC-3’, iNOS (Forward: 5’-TAGGCACCTTTGTGGTGCAG-3’ and Reverse: 5’-CGGCTCTGAATCTTCTATCC-3’) genes using Syber Green PCR Master Mix technology.

All experiments were performed by ^TM^QuantStudio 3 (Applied Biosystems, Life Technologies Corporation) and software QuantStudio. Gene expression was analyzed by Cycle threshold (Ct) comparative technique (2^-^ΔΔCt) after normalization between genes of interest and the housekeeping (β-actin). The experiments were performed in triplicate. For immunohistochemistry, briefly, knee samples were treated with anti-iNOS (ab3523, 1:500) and biotinylated secondary antibodies DAKO LSAB (DAKO, Glostrup, Denmark) incubation with horseradish peroxidase. Knee samples were washed, incubated with streptavidin biotin-peroxidase complex and 3,3’-diaminobenzidine (DAB) solution. The counting of positive immunoreactive cells (number of cells in 10^4^ μm^2^) was performed by ImageJ (NIH/USA, free program) as described before by our group^13^.

### Statistical analysis

Graph Prism 5.0 was used to calculate the arithmetic mean and standard deviation (SD). The values of 2^-^ΔΔCt and immunohistochemistry were statistically analyzed using analysis of variance (ANOVA) test and post-hoc Tukey’s test, and it was set the significance level of 5% (p < 0.05). All data was normalized before the statistical analysis.

## Results

### Morphological analyses

**Figure 1:**
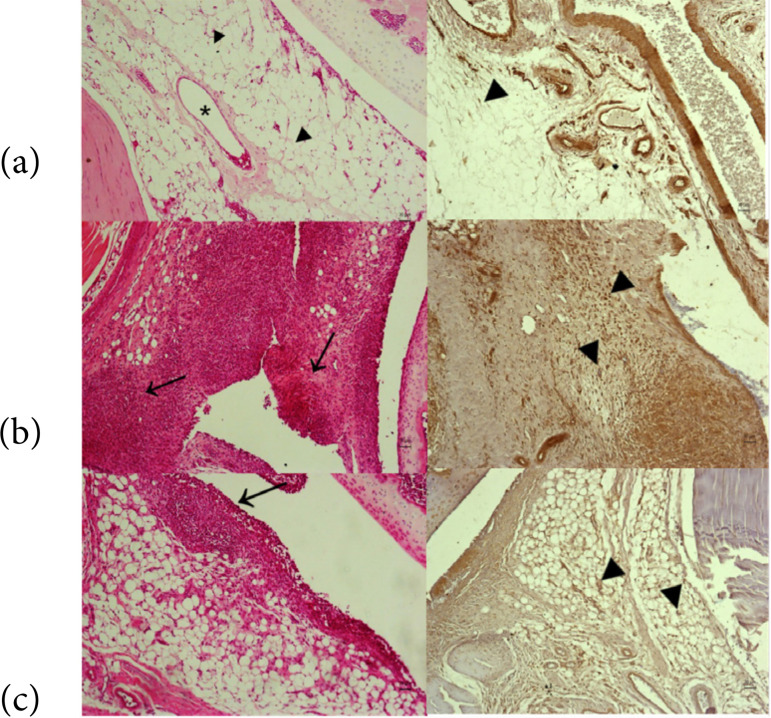
Morphological aspects of Wistar Rats sinovia stained with hematoxylin and eosin (left column; arrowhead-normal synoviocytes, asterisk-blood vessel, arrow-inflammatory process) and immunohistochemistry (right column, inducible nitric oxide synthase antibody stained with 3,3’-diaminobenzidine; arrowhead-positive cells). **(a)** Control; **(b)** sham; **(c)** arthritis and photobiomodulation.

Control group showed normal synovial structure and preservation of membrane, blood vessels, and synoviocytes. It was observed an increase in the thickness of the synovial lining epithelium and inflammatory infiltrate, in addition to proliferation of fibroblastic cells, neoangiogenesis, and tissue fibrosis in sham. Animals submitted to PBM showed reduction in the inflammatory infiltrate associated with an increase in medium-sized blood vessels and reduction of lining epithelium thickness (Fig. 1).

### Gene expression

**Figure 2:**
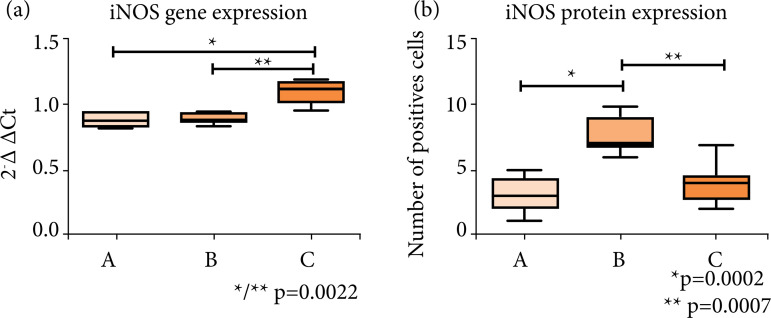
Statistical analysis after data normalization between the experimental groups (mean±SD), significant level of 5% (p < 0.05). **(a)** Values of inducible nitric oxide synthase (iNOS) gene expression analyses by real time polymerase chain reaction after reverse transcription, significant differences were observed between AxC and BxC. **(b)** iNOS protein expression by immunohistochemistry, significant differences were observed between AxB and BxC. **(A)** Control; **(B)** sham; **(C)** arthritis and photobiomodulation.

Gene expression analysis after normalization between iNOS gene and β-actin (housekeeping) produced ΔCt values (mean ± SD) that showed significant differences in iNOS expression between sham (0.883 ± 0.040) and PBM (1.095 ± 0.089), p = 0.0022, and control (0.874 ± 0.052) and PBM groups, p = 0.0022 (Fig. 2a).

### Immunohistochemistry

Immunohistochemical analyses (mean ± SD) showed significant differences between control (3.1 ± 1.2) and sham (7.6 ± 1.3), p = 0.0002, and sham and PBM groups (3.9 ± 1.5), p = 0.0007 (Figs. 1b and 2b).

## Discussion

In our study, we used a female animal model, since the rate of rheumatoid arthritis in women is higher than in men^14^. The induced arthritis is characterized as acute, since the analyses were performed over seven days, since from the 14th day, in this model of RA induced by Zymosan, the cellular and morphological profile is equivalent to the chronic condition^15^. The intra-articular application of Zymosan promotes the induction of arthritis through the stimulation of nociceptors. These nociceptors are in periarticular tissues and, when stimulated, increase local permeability, leading to marked edema and major cell degradation due to the acute inflammation caused^16^.

From a morphologic and functional point of view, acute inflammation is characterized by exudative phenomena leading to an accumulation of fluid (edema), leukocytes (neutrophils and mast cells), erythrocytes, and pro-inflammatory factors^12^. These were the changes found in the sham animals in our study.

Low-level laser PBM is emerging as an alternative to the use of drugs. PBM with low-level laser has photochemical, photophysical, and photobiological mechanisms, particularly via cytochrome C oxidase, a terminal enzyme in the mitochondrial electron transport chain that causes changes in the biological system by increasing adenosine triphosphate (ATP) production^17^^,^ 18. In our study, it is believed that mitochondrial pathways are stimulated after PBM, which is related to the increase in iNOS expression in synovial cells.

As a result of the increase in ATP, the cells undergo biostimulation, promoting possible therapeutic effects such as morphodifferentiation and cell proliferation, tissue neoformation, increased local microcirculation through angiogenesis, anti-inflammatory and analgesic potential^18^.

In our study, morphological changes proved the induction of the inflammatory process, and the fact that iNOS was studied is justified because the gene and protein are generally not expressed in cells and tissues at rest, but can be synthesized after cell activation, as in the inflammatory process itself, and because of the mechanism exerted by PBM with LLLT^19^.

In laser therapy, nitric oxide plays an important role in balancing the antioxidant system, as it is an important inter and intracellular messenger involved in various physiological and pathophysiological conditions. Studies show that it is possible to observe the effects of nitric oxide in low power laser treatment, as nitric oxide is part of the radiation-induced mesenteric process, causing arteriolar vasodilation and consequently an increase in blood flow, demonstrating antioxidant balance, improving inflammation, and reducing local swelling^20^^,^ 21.

Research using cDNA microarrays has shown that red light irradiation regulates the expression of genes in fibroblasts. Those genes related to antioxidant and mitochondrial energy metabolism are expressed after irradiation. In addition to various other regulatory functions of the organism, nitric oxide has been also recognized as a potential signal that controls cellular respiration, and nitric oxide flow is extremely important in reactions within cells^20^.

In the bloodstream, nitric oxide is involved in the coagulation cascade, and in normal physiological functions it plays a role as a vascular modulator, also dilating the vessel and increasing blood flow due to its ability to relax smooth muscle. If there is a decrease in nitric oxide, there is also the appearance of vasoconstriction. On the other hand, if there is an increase in nitric oxide, there is the production of pronounced vasodilation and shock, with reduction in platelet activity, and homeostasis is impaired^22^.

Studies have shown that LLLT is active in reducing iNOS production when lipid peroxidation is reduced. Cyclooxygenase-2 gene expression is lower and iNOS concentrations are lower when PBM with λ = 808 nm and energy of 1.4 J were used, or in increasing iNOS production between 30 min and one day after irradiation^23^^,^ 24.

Moriyama et al.^22^ analyzed the effects of LLLT at λ = 690 nm, 25 mW power and 5 J energy in a single exposure on iNOS production, observing inhibition of production. When compared to λ = 905 nm, there was greater expression of iNOS, which corroborates our findings using the same chromophore in infrared light. In our study, we observed an increase in the expression of iNOS genes in the irradiated group, while protein expression was reduced. It is suggested that iNOS activation pathways are related to an increase in oxidative balance through an increase in systemic antioxidant enzymes, which can reduce protein levels.

These effects have already been described by our research group, which evaluated the effects of the laser on systemic oxidative stress levels using nitric oxide dosages, which showed reduction when the laser was applied in the same parameters as those used in this study^12^. The hypothesis of antioxidant effects has already been proven by a study that evaluated an increase in antioxidant enzymes such as superoxide dismutase and catalase andreduction in oxidative molecules such as glutathione peroxidase and malondialdehyde after the application of infrared laser in patients with rheumatoid arthritis^25^.

As limitations of our study, lack of information about the role of iNOS in acute inflammatory response with zymosan-induced arthritis was observed, and it was not analyzed the antioxidant enzymes in this study, that differs between human and experimental models.

## Conclusion

PBM upregulated iNOS gene expression, however downregulated protein expression compared to sham, which suggests a secondary mitochondrial stimulation that can promote an increase of antioxidant enzyme balance or decrease of neutrophils migration that can be observed in morphological analyses with higher inflammatory infiltration without treatment. Further studies can be conducted to elucidate the role of mitochondria in inflammatory reactions and cell signaling in inflammation and arthritis.

## Data Availability

The data will be available upon request.
